# Electrostatic Field Enhanced Photocatalytic CO_2_ Conversion on BiVO_4_ Nanowires

**DOI:** 10.1007/s40820-021-00749-6

**Published:** 2021-12-06

**Authors:** Shuai Yue, Lu Chen, Manke Zhang, Zhe Liu, Tao Chen, Mingzheng Xie, Zhen Cao, Weihua Han

**Affiliations:** 1 Key Laboratory for Environmental Pollution Prediction and Control of Gansu Province, College of Earth and Environmental Sciences, Lanzhou University, Lanzhou, 730000 People’s Republic of China; 2 School of Physical Science and Technology, Lanzhou University, Lanzhou, 730000 People’s Republic of China

**Keywords:** Photocatalysis, CO_2_ reduction, Electrostatic field, BiVO_4_ nanowires

## Abstract

The recombination loss of photo-carriers in photocatalytic systems fatally determines the energy conversion efficiency of photocatalysts. In this work, an electrostatic field was used to inhibit the recombination of photo-carriers in photocatalysts by separating photo-holes and photo-electrons in space. As a model structure, (010) facet-exposed BiVO_4_ nanowires were grown on PDMS-insulated piezo-substrate of piezoelectric transducer (PZT). The PZT substrate will generate an electrostatic field under a certain stress, and the photocatalytic behavior of BiVO_4_ nanowires is influenced by the electrostatic field. Our results showed that the photocatalytic performance of the BiVO_4_ nanowires in CO_2_ reduction in the negative electrostatic field is enhanced to 5.5-fold of that without electrostatic field. Moreover, the concentration of methane in the products was raised from 29% to 64%. The enhanced CO_2_ reduction efficiency is mainly attributed to the inhibited recombination loss of photo-carriers in the BiVO_4_ nanowires. The increased energy of photo-carriers and the enhanced surface absorption to polar molecules, which are CO in this case, were also play important roles in improving the photocatalytic activity of the photocatalyst and product selectivity. This work proposed an effective strategy to improve photo-carriers separation/transfer dynamics in the photocatalytic systems, which will also be a favorable reference for photovoltaic and photodetecting devices.

**Supplementary Information:**

The online version contains supplementary material available at 10.1007/s40820-021-00749-6.

## Highlights

- BiVO_4_ nanowires with exposed (010) facets were deposited on the piezoelectric transducer piezo-substrate.
- CO_2_ conversion rate was significantly improved by 5.5-time in an electrostatic field generated by applying a stress on the piezo-substrate.
- The mole percentage of methane in products was increased from 29% to 64%.
- The improved performance was attributed to the facilitated photo-carriers separation/transfer, as well as the enhanced adsorption of polar molecules.

**Supplementary Information:**

The online version contains supplementary material available at 10.1007/s40820-021-00749-6.

## Graphical abstract

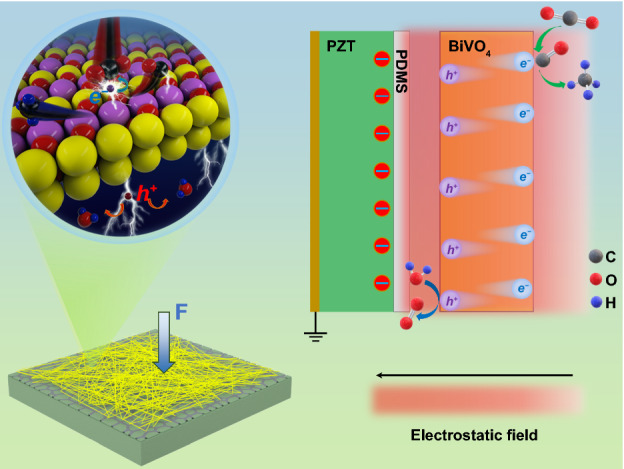

## Introduction

Excess CO_2_ emissions brought by the rapid development of global industry have resulted in serious environmental and ecological problems [1, 2]. Photocatalysis based on semiconductors provides an ideal way to reduce the CO_2_ concentration in the atmosphere, along with the generation of valuable product, and has been drawn more and more attention [3–5]. As the key factor to determine the photocatalytic performance, an ideal photocatalyst should be stable and superior in light absorption and redox activity, which depends on the band structure. According to previous reports, an optimal band-gap for photocatalysis is about 2.0–2.4 eV [6–8]. Among the reported photocatalysts, metal oxides with narrow band-gap, such as BiVO_4_, Fe_2_O_3_, and WO_3_, meet the above requirements well. The main barriers to their applications are the short diffusion length of photo-carriers and weak reductivity of photo-electrons [9–11]. Most of the photo-carriers have been recombined before reaching the photocatalyst surface. Even if some of them successfully reach the photocatalyst surface, their energy has been partially dissipated, which is easily trapped by surface defects. For instance, the hole diffusion length in BiVO_4_ is smaller than 50 nm [12, 13], which limits the thickness of photocatalytic layers to guarantee as many photo-carriers as possible to reach the photocatalyst surface. However, the thinner the photocatalytic layer, the less light utilization for less light absorption in the thin film layer. Moreover, the conduction band (CB) of BiVO_4_ is ~ 0 V *vs.* SHE is weak for CO_2_ reduction [14], which only meets the minimum requirement despite the overpotential.

As known, an electrical potential gradient in photocatalyst works as a driving force for photo-carriers separation and transfer [15]. Photo-holes are driven to one side of the photocatalytic film, while photo-electrons to the other side, and thus realizing spatial separation of positive and negative charges. The recombination probability is reduced and significantly improves the effectivity of photo-charges. Potential gradient in photocatalyst is usually realized by building a type-II or Z-scheme heterojunction [16, 17]. Type-II heterojunction was commonly formed by coupling two n-type semiconductors. The CB and valence band (VB) of one semiconductor (SC-I) are slightly lower than those of the other semiconductor (SC-II) [18, 19]. A potential gradient is built when the two semiconductors contact together. The SCI-I acts as an electron sink, while SC-II acts as a hole-sink. Although the photogenerated holes and electrons can be effectively separated in space and inhibit their recombination, the redox activity is also reduced due to the redox reactions that occurred under lower potentials [20–22]. So, the separation and transfer efficiency of photo-carriers is improved by sacrificing its redox activity in this case.

Compared with type-II heterojunctions, Z-scheme heterojunctions also have a staggered band-gap structure, but the CB of SCI-I is located near the VB of SC-II [23]. Photo-electrons in SC-I tend to recombine with photo-holes in SC-II and thus leave high energy holes in SC-I and high energy electrons in SC-II. The distinct charge migration path in Z-scheme heterojunction successfully separates photo-carriers by maintaining their high redox activity, however, half of the photo-carriers have been consumed [24–26]. The high redox activity of photo-carriers is maintained by sacrificing its quantity. Despite the achieved progresses in these manners, the potential gradient realized with heterojunctions is a double-blade sword. It is still challenging to substantially address the separation and transfer issues of photo-carriers without sacrificing their activity and quantity due to their limited CB and VB potential difference between semiconductors and charge transfer ability of the semiconductors.

Here, we proposed to use an external applied electrostatic field to provide the potential gradient in photocatalysts. The electrostatic field was generated by applying a stress on a piezo-substrate from the top of the photocatalysts. In this work, (010) facet-exposed BiVO_4_ nanowires were selected as photocatalysts. A piece of polarized polycrystalline PZT wafer was used as the piezo-substrate. Once a stress is applied on the piezo-substrate, the BiVO_4_ nanowires will be immersed in an electrostatic field. A thin layer of polydimethylsiloxane (PDMS) was used to electrically isolate the photocatalyst and the substrate. The results show that the negative electrostatic field generated on the top of the PZT substrate significantly improves the photocatalytic performance of BiVO_4_ nanowires in CO_2_ reduction. The activity was improved by 5.5 times after the electrostatic field was applied. The content of methane in products was also increased from 29 to 64%. This work provides an effective and comprehensive strategy to promote the photocatalytic performance of photocatalysts by enlarging potential gradient with the electrostatic field, which is almost no additional power input.

## Experimental Methods

### Preparation of (010) Facet-exposed BiVO_4_ Nanowires

The (010) facet-exposed BiVO_4_ nanowires were synthesized by a template-ion exchange method [27]. Typically, a mixture containing 1 mmol of V_2_O_5_, 2 mmol of Na_2_SO_4_ and 30 mL of deionized water was placed in a Teflon-lined autoclave with a capacity of 50 mL. The autoclave was kept at 180 °C for 24 h, and Na_2_V_6_O_16_·3H_2_O nanowire templates were obtained after cooling down. Then, 132 mg of the above templates were dispersed into 40 mL of solution consisting of nitric acid, ethanol and water (V_nitric acid_/V_ethanol_/V_water_ = 2:4:1), and 582 mg of Bi(NO_3_)_3_·5H_2_O (1.2 mmol). Then, 0.3 g of C_18_H_29_NaO_3_S (SDBS) was added, and the mixture was adjusted to pH 6.5 with 1 M NaOH. The mixed solution was transferred into a 50 mL Teflon-lined autoclave for hydrothermal reaction (120 °C for 24 h). After being cooled down, (010) facet-exposed BiVO_4_ nanowires were obtained and denoted as BVO-NWs.

### Fabrication of BiVO_4_ NWs/PDMS/PZT Device Structure

**Scheme 1:**
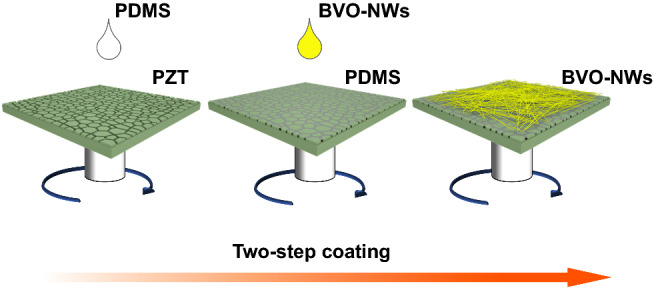
Depositing of BiVO_4_ nanowires on PZT piezoelectric substrate

The composite of BiVO_4_ NWs/PDMS/PZT device structure was fabricated through a 2-step process (Scheme 1). Prior to fabricating, the commercial PZT wafer was pretreated with dilute nitric acid to remove the silver coating covered on the surface (Fig. S1a-f). PDMS prepolymer and curing agent (Sylgard 184, Dow Corning) were thoroughly mixed in a mass ratio of 10:1. First, an insulating layer of PDMS was coated on a pretreated PZT wafer by spin-coating the above mixture at the speed of 6000 r s^−1^ (Fig. S1g-i). Then, it was kept at 80 °C for 1 h for cross-linking. Afterward, the BVO-NWs were coated by spin-coating 100 μL of mixture made up of 4.85 mg of BVO-NWs and 100 μL of ethanol at the speed of 2000 r s^−1^. The obtained composite was dried at 60 °C in air and denoted as BVO-NWs/PDMS/PZT.

### Characterizations

The micro-morphologies were observed by means of a scanning electron microscope (SEM, Hitachi S-4800) and transmission electron microscope (TEM, FEI Tecnai F30). The thickness of the film was determined by means of an atomic force microscope (AFM, Asylum Research MFP-3D). The crystal structures were investigated by X-ray diffraction (XRD) patterns (Philips X’pert Pro, Cu Kɑ, 0.15406 nm) and Raman spectra (JY-HR800, 532 nm laser). XPS (Kratos-AXIS ULTRA DLD) and EDX spectra equipped on the TEM were employed to explore the surface chemical composition. Optical properties were studied by means of a UV–Vis spectrometer (TU-1901). The surface photovoltage (SPV) spectra were recorded on a home-built apparatus to study the charge carrier properties [28]. Photoluminescence emission spectra at 340 nm excitation were recorded on a ZLX-PL-I instrument.

Photoelectrochemical (PEC) measurements were carried out on a CHI660E electrochemical workstation. The electrolyte is 0.5 M sodium sulfate and the illumination source is a 500 W Xenon lamp with a cutoff filter (*λ* > 420 nm). A piece of Pt foil was used as the counter electrode and a saturated KCl Ag/AgCl electrode as reference. For EIS measurements, 10 mV and 1 V were set as the amplitude and applied bias, respectively. Incident photon-to-current conversion efficiency (IPCE) and apparent quantum yield (AQE) spectra and the photo-carriers diffusion lengths were calculated and the corresponding details were shown in Supporting Information.

The PZT substrate, BVO-NWs/PDMS/PZT were assembled into two-electrode structures with copper foil to carry out the piezoelectric measurements (Fig. S2). A periodical stress was applied by a magnetic vibration platform, and a 50 W LED lamp with a wavelength of 450 nm was used as the incident light source. The signal was captured by a CHI660E electrochemical workstation.

### Numerical Simulation and DFT Calculation

The electrostatic field was simulated by means of COMSOL Multi-physics software (version 5.4). The direction of the applied stress is from the top to the bottom, and it is 0.2 N in intensity. The detailed parameters of PZT during simulation include area, piezo constant (d_33_), relative dielectric permittivity (ε_r_) and Young’s modulus, which are 3.14 cm^2^, −1.7 × 10^–10^ C N^−1^, 1700 and 36 GPa, respectively.

The binding energies of the CO_2_, CO and CH_4_ on the defective (010)-BiVO_4_ slab were evaluated using the density functional theory (DFT) calculations through the VASP package. We used the VASP-sol method [29, 30] to explore the variation of the binding energy with the applied voltage. All the calculations were performed at the Perdew-Burke-Ernzerhof (PBE) [31] level of exchange function with U parameters to constrain the d electrons of V [32], and the vdW interaction was described using the Grimme’s correction [33, 34]. The Brillouin zone was sampled at the 3 × 3 × 1 k-points. The structural optimization was assumed to be converged if the force is smaller than 0.001 Ev Å^−1^.

The adsorption energies of CO_2_, CO and CH_4_ on the BiVO_4_ layer are calculated from the Equation: E_abs_ = E_1_-E_2_-E_3_ , where E_1_ is the total energy of BiVO_4_ with the adsorbed gas molecule, E_2_ is the energy of the isolated BiVO_4_ layer, and E_3_ is the energy of a CO_2_, CO or CH_4_ molecule in the gas phase.

### Photocatalytic Activity Evaluation

Photocatalytic CO_2_ reduction was carried out to evaluate the photocatalytic activity in a home-built apparatus, which is a steel reactor equipped with a quartz window and a 300 W Xenon lamp. In the steel reactor, 3 mL of water was placed and plenty of pure CO_2_ was supplied through water and reached ambient pressure. The prepared sample was placed above the water by means of a holder, and a piece of special quartz glass was covered on its surface to provide the desired stress (Fig. S3). After being irradiated, the concertation and composition of products were detected by a gas chromatograph (GC-7900 with TCD, Perfect Light, Beijing) at regular intervals.

## Results and Discussion

### Structural Features

**Fig. 1:**
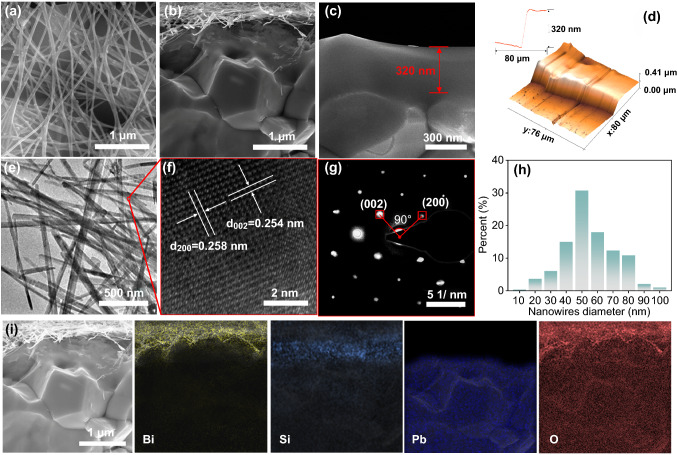
Microstructures of samples. **a-c** SEM images of BVO-NWs/PDMS/PZT and PDMS/PZT. **d** AFM image which reflects the thickness of the PDMS layer. **e–g** TEM images and SAED pattern of BiVO_4_ nanowires. **h** Diameter distribution of nanowires. **i** EDS elemental maps of BVO-NWs/PDMS/PZT

The micro-morphologies are shown in the SEM and TEM images. The top-view and cross-sectional view of the device were shown in Fig. 1a, b. BiVO_4_ nanowires were randomly distributed on the insulating PDMS layer with a thickness of about 320 nm (Fig. 1c, d). The BiVO_4_ nanowires are long and thin, with a length of several micrometers and a diameter of tens of nanometers. (Fig. 1e). The clear and orderly lattice fringes shown in Fig. 1f implies that the BiVO_4_ nanowires have high degrees of crystallinity. There are two lattice spacings of 0.258 nm and 0.254 nm which correspond to the (200) and (002) planes of the BiVO_4_ monocline scheelite [35, 36]. From the SAED pattern (Fig. 1g), it shows that the BiVO_4_ nanowires are single crystals. The nanowire structure of BiVO_4_ was constructed through an ion exchange process, and its unique morphology was derived from the Na_2_V_6_O_16_·3H_2_O nanowire templates (Fig. S4).

From the SAED pattern, the angle between the (002) plane and (200) plane is 90°, indicating the BiVO_4_ nanowires possess a highly exposed (010) facet. According to the diameter distribution, the diameters of BiVO_4_ nanowires are ranged from 20 to 100 nm, and the average diameter is about 50 nm (Fig. 1h). The EDX spectra and elemental maps are shown in Figs. S5 and 1i further prove the 3-layer structure of a constructed composite of PZT substrate and BiVO_4_ nanowires.

**Fig. 2:**
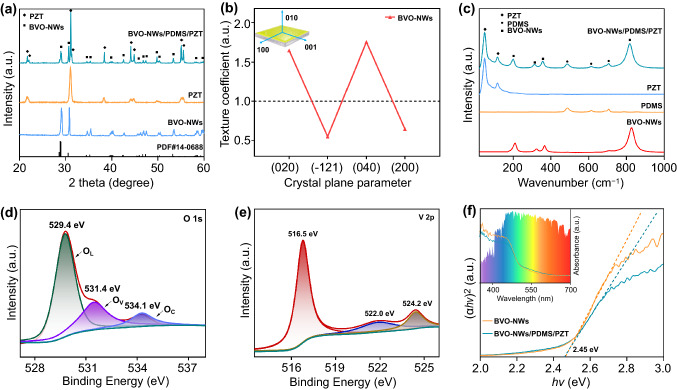
Structural and surface chemical compositions. **a, b** XRD patterns and corresponding TC curves. **c** Raman spectra. **d, e** High-resolution XPS spectra of BiVO_4_ nanowires. **f** Tauc-plots, the inset is the corresponding UV–Vis absorption spectra

The phase composition was studied by XRD and Raman spectra. As shown in Fig. 2a, the diffraction peaks of nanowires well agree to the standard PDF card of monoclinic BiVO_4_ (No. 14–0688). It is noted the diffraction peak at 30.5° ascribed to (040) facets is remarkably higher than expected, indicating a specific facet exposure, which is consistent with the TEM results. For BVO-NWs/PDMS/PZT composite, the diffraction peaks from PZT substrate still possess considerable intensities. It is mainly attributed to the thin thickness and reticular structure of BiVO_4_ layer which could only be observed in the SEM image. Figure 2b shows the calculated texture coefficients (P) of some facets for BVO-NWs. The BVO-NWs sample shows a high P-value corresponding to (010) facets of 1.75 compared with the bulk BiVO_4_ sample, demonstrating a high exposure ratio of (010) facets. The presence of PDMS layer decorated on a PZT substrate was verified by means of Raman spectra (Fig. 2c), in which there are peaks at 486, 613, and 707 cm^−1^ ascribed to PDMS [37, 38]. In addition, for BVO-NWs sample, the position of the peak indexed to symmetric V–O bond stretching mode is 816 cm^−1^, while that for bulk BiVO_4_ is 811 cm^−1^ (Fig. S6). The slight shift implies the oxygen defects on BVO-NWs surface [39, 40].

The surface chemical composition was investigated by performing XPS measurements. From the survey spectrum (Fig. S7a), the BiVO_4_ nanowires are composed of Bi, V, and O elements, agreeing with the results of EDX spectra. The asymmetric O 1 s peaks shown in Fig. 2d could be fitted into three components, which are located at 529.4, 531.4, and 534.1 eV. They are derived from the lattice oxygen (O_L_), oxygen vacancy (O_V_), and adsorbed oxygen (O_C_), respectively [41, 42]. Such a result indicates that there is a certain amount of oxygen defects in the prepared BiVO_4_ nanowires. By comparing the areas, the percentage of O_V_ was determined to be 30.4%, while those of O_L_ and O_A_ are 57.6% and 12%, respectively. The oxygen defects have influences on the adjacent V and Bi atoms in the lattice. In Fig. 2e, a peak at 522.0 eV could be observed, suggesting there are some V^4+^ in the BiVO_4_ nanowires [43]. Moreover, the binding energies of Bi 4f are located at 159.0 and 164.3 eV, a little lower than those of the common BiVO_4_ (Fig. S7b) [44]. As the Tauc-plots curves shown in Fig. 2f, the band-gap of BiVO_4_ nanowire is 2.45 eV, the same as that of common BiVO_4_ particles [45]. It is worth noting that the BVO-NWs sample exhibits a strong visible light absorption due to its intertwined mesh structure.

### Piezo Properties and Influences

**Fig. 3:**
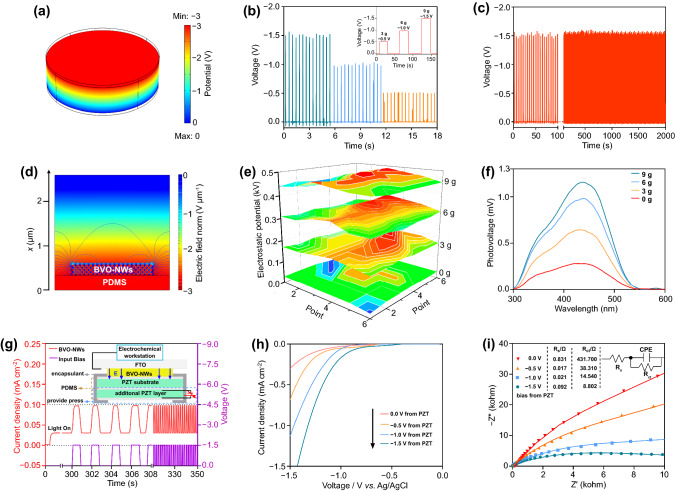
Piezoelectric property of PZT and its influences on photogenerated charge carriers. **a** COMSOL simulation of the piezo-potential distribution in PZT. **b** Open-circuit voltages of PZT under different stresses. **c** Long-term piezoelectric stability of PZT substrate. **d** COMSOL simulation of the influence range of piezo-electrostatic field. **e** Surface electrostatic potential of BiVO_4_ nanowires loaded on PZT substrate. **f** Surface photovoltage spectra of BVO-NWs/PDMS/PZT. **g** *I-t* curve under the influence of electrostatic field from PZT substrate. **h, i** *I-V* curves and Nyquist plots in CO_2_-bubbled system

The electrostatic field generated with the PZT piezo-substrate was studied by means of COMSOL simulation and an actual piezoelectric test. Under a vertical downward stress of 20 g, the PZT substrate would be polarized. Negative charges aggregate at the top-surface, inducing a downward electric field in the upper half-plane, and reverse on the other side. The potential difference between the top side and bottom side is about -3 V (Fig. 3a). The output is very sensitive to the applied force, and the intensity would get enlarged as the increase of the applied force (Figs. 3b and S8). The output of PZT substrate decays rarely in 500 cycles under a periodic stress of 4 s (Fig. 3c). Moreover, the generated voltage could be kept constant in air for more than 8 h under constant stress (Fig. S9), which is mainly attributed to the low electrical leakage of PZT [46]. The simulation results shown in Fig. 3d indicate that the negative potential at the top of PZT could generate a negative electrostatic field, of which impact could reach 1 micro-meter in air even in the presence of PDMS layer. The loaded BiVO_4_ layer with a thickness of 250 nm and the relative permittivity of 68 is completely under the influence of the piezoelectric field. The action of the electrostatic field was further proved by the enhanced surface electrostatic potential on the BiVO_4_ layer under stress (Fig. 3e).

The photogenerated charge carrier properties were studied to explore the influences of the electrostatic field. Figure 3f shows the surface photovoltage spectra of BVO-NWs/PDMS/PZT composite under different stresses. As an N-type semiconductor, BiVO_4_ would show a positive SPV signal under light irradiation, of which generation depends on the photogenerated charge separation [47]. After being pressed, the SPV signal of the composite gets enhanced remarkably, and the increment is proportional to the strength of the applied force. It indicates the electrostatic field facilitates photogenerated charge separation, which is further proved by the reduced PL signal (Fig. S10).

The electrostatic field can be generated by applying a stress on the piezo-substrate. In other words, the PEC reaction can be controlled by adjusting the intensity of the applied stress, so as to reflect the influence of the electrostatic field. To rationally control the PEC reaction rates, another PZT wafer with top and bottom electrodes was fixed to the bottom of the piezo-substrate (inset of Fig. 3g). When a voltage is applied to the bottom PZT wafer, the piezo-substrate will be compressed or released due to the electrostrictive effect of the bottom PZT wafer. In such an architecture, the applied stress on the piezo-substrate can be rationally and accurately controlled by the voltage applied on the bottom PZT wafer. The piezo-potential generated on the piezo-substrate varies with the bias voltage applied on the bottom PZT wafer as recorded in Fig. S11. From Fig. 3g, the photocurrent from BiVO_4_ nanowires was increased and decreased with the DC bias applied on the bottom PZT wafer. It indicates that the separation and transfer of photo-carriers have been significantly improved, which is consistent with the SPV spectra. Figures 3h and S12 show the PEC CO_2_ reduction curves under different additional DC biases. The application of DC bias not only enhanced the photocurrent density but also reduced the onset potential. It demonstrates that the piezo-electrostatic field could promote the separation and transfer of photo-carriers, and their energy was also increased. According to EIS Nyquist plot spectra shown in Fig. 3i, the radii of the capacitive arc get reduced under the piezo-electrostatic field. In general, a small radius represents small charge transport resistance. By fitting the curves, it is reasonable to conclude that the reduction is mainly resulted from the remarkably reduced R_ct_, which is the charge transport resistance between the nanowires [48].

### Activities for CO_2_ Reduction

**Fig. 4:**
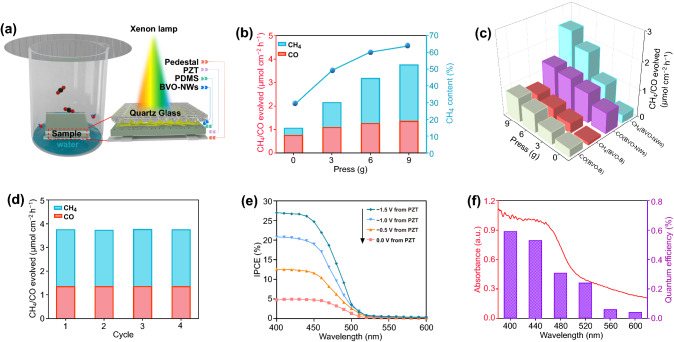
Photocatalytic CO_2_ reduction. **a** Schematic diagram of the CO_2_ reduction device. **b** Reduction of CO_2_ over BVO-NWs/PDMS/PZT under different stresses. **c** Comparison of activities of BVO-NWs and bulk BiVO_4_. **d** Photocatalytic performance of BVO-NWs/PDMS/PZT in 4 cycles. **e, f** IPCE spectra and wavelength dependence of AQE of BVO-NWs/PDMS/PZT

To evaluate the photocatalytic activity, CO_2_ reduction reactions were carried out in a home-built installation as shown in Fig. 4a. According to the mass of covered quartz glass, a desired negative bias would be provided by the PZT substrate (Fig. S9). From Fig. 4b, after being irradiated for 8 h, a certain amount of product consisting of CO and CH_4_ was detected over BiVO_4_ nanowires without the covering of quartz glass. It is superior to the many reported BiVO_4_ samples with other morphologies (Table S2), which may be due to the small characteristic size and single-crystal structure. When the BiVO_4_ nanowires were covered by the quartz glass with a certain mass, both the CO and CH_4_ generation rates were promoted. The heavier the quartz glass, the higher the activity. The activities for CO and CH_4_ generation could reach 1.37 and 2.41 μmol cm^−2^ h^−1^ under the stress of a 9 g quartz glass. Moreover, with the promotion of activity, the CH_4_ concentration in products was increased from 29 to 64% (Fig. 4b, c). The activity of bulk BiVO_4_ was also promoted by the electrostatic field, but it is lower than that of BiVO_4_ nanowires, which is mainly due to the inferior charge carrier separation (Fig. S13). The photocatalytic performance of BiVO_4_ nanowire under stress decays rarely in 4 cycles, indicating a good stability (Fig. 4d). The IPCE is an important parameter to evaluate the photocatalytic activity. As the IPCE spectra were recorded in a CO_2_ saturated solution (Fig. 4e), the IPCE values for BiVO_4_ nanowires were enhanced from 4.74 to 26.99% at 400 nm under stress, meanwhile, the apparent quantum yield in the same condition could reach 0.59% (Fig. 4f).

### Mechanism

**Fig. 5:**
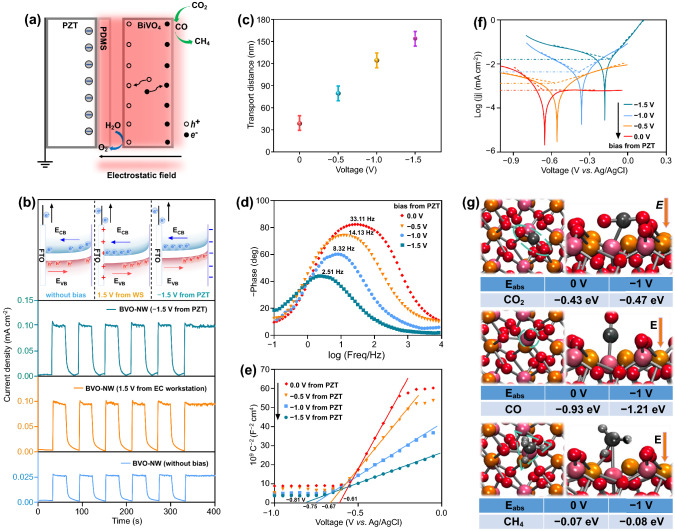
Influence mechanism of piezo-electrostatic field.** a** Schematic diagram of influences of piezo-electrostatic field on charge carriers behavior and CO adsorption.** b** *I-t* curves under different conditions and the inset show the features of band structures and charge carrier transport behaviors. **c** Calculated photogenerated charge carriers diffusion lengths. **d, e** Bode phase plots and Mott-Schottky plots. **f** Tafel curves. **g** Adsorptions of CO_2_, CO, and CH_4_ on BiVO_4_ layer and their corresponding adsorption energy

Based on the above investigation, we can conclude that the photocatalytic activities of BiVO_4_ nanowires for CO_2_ reduction could be enhanced by applying an electrostatic field. As mentioned in the Introduction section, when a photocatalyst is immersed in an electric field, a potential gradient will be built, and the transport behavior will be demonstrated by the potential distribution. As shown in Fig. 5a, once a stress is applied on the piezo-substrate, the right side of the piezo-substrate generates a piezo-electric field. The generated piezo-electric field creates a potential gradient in the photocatalyst. Photo-electrons move to the right side driven by the electric force and accumulate at the top-surface photocatalyst, waiting to take reduction reactions. While photo-holes migrate to the other side (left side in the figure) of the photocatalyst and undergo an oxidation reaction at the bottom with CO_2_ penetrated to the porous structure. Because the piezoelectric wafer was insulated by the PDMS layer, piezo-electric charge cannot be neutralized by the photo-holes from the photocatalyst (here is BiVO_4_). It is an electrostatic field, and there is almost no power input if we neglect the leakage current. There are three effects of the electrostatic field on the transport behavior of photo-carriers. First, the potential gradient provides a driving force for photo-holes and photo-electrons separation and transfer, which significantly inhibits their recombination. Second, the photo-carriers become more energetic after reducing their electric potential, which increases their activity in escaping from surface defect trapping and taking redox reactions. Third, the electric field will also facilitate the adsorption of polarized molecules or groups on the photocatalyst surface, which highly promotes the product selectivity.

A series of experiments were carried out to prove the mechanism. As an N-type semiconductor, the bands of BiVO_4_ would bend upward near the interface of BiVO_4_ and electrolyte, thus exhibiting a positive photocurrent without a bias during PEC measurements (Fig. 5b). The photocurrent is small since the influence of band bend is limited. When a positive bias for the workstation is applied to the FTO side of the electrode, the induced opposite transport of electrons and holes is promoted, resulting in a remarkably enhanced photocurrent [49]. The negative electrostatic bias applied to the catalyst side plays similar roles as the positive bias from the workstation, indicating the negative piezo-electrostatic field also promotes the opposite transport of electrons and holes. The inductive effect of the electrostatic field obviously increases the diffusion length of photogenerated charge carriers. As shown in Fig. 5c, the initial charge 39.3 nm length of BiVO_4_ is no more than 50 nm, which is consistent with the results reported [50]. In negative electrostatic potential of −1.5 V, it could extend to 153.7 nm. Meanwhile, the lifetime of the charge carrier which could be calculated by equation τ_e_ = 1/2πf_max_ was prolonged from 4.8 to 63.4 μs (Fig. 5d and Table S3), as well as its concentration is enhanced (Table S4).

The enhancement of photo-carriers energy under different piezo bias is reflected in Fig. 5e. It is clear the negative piezo-electrostatic field made the flat-band potential more negative. When the piezo bias is −1.5 V, the corresponding elevation is ~ 0.2 eV. The photo-electrons are therefore more reductive to react with CO_2_, which is proved by the increased exchange current and decreased required potential in Tafel curves (Figs. 5f and S14). The absorption of molecules was studied by means of DFT calculations, of which results are shown in Figs. 5g and S15. For O vacancy-rich BiVO_4_ nanowires, CO_2_ molecules would be thermodynamically absorbed on V atom adjacent to the O vacancy. As nonpolar molecules, the adsorption energies of CO_2_ and CH_4_ change rarely when an external electrostatic field is applied. However, it is different for CO, of which adsorption energy is enhanced by ~ 30%. It is suggested the CO molecule would absorb BiVO_4_ more tightly under the influence of the electrostatic field. According to literature, promoted adsorption of CO will lead to a preferred generation of CH_4_ [51, 52].

## Conclusions

In summary, we have verified the electrostatic effect on photocatalysis, which significantly improves the photocatalytic performance of photocatalysts in energy conversion efficiency and redox activity. As a model structure, BiVO_4_ nanowires are porously distributed on PDMS-insulated piezo-PZT substrate. Once a stress is applied from the top of PZT piezo-substrate, the generated piezoelectric field will serve as a driving force for photo-carriers separation and transfer, which also facilitate the occurrence of redox reactions by promoting the energy of photo-carriers and reaction probability through enhancing surface absorption to polar molecules/groups. Such an electrostatic enhancement can be used in a variety of fields including photocatalysis and similar fields. Our results show that when a stress was applied by a loading mass of ~ 9 g, the reduction efficiency was enhanced by 5.5-time more than without stress, and the content of CH_4_ in products was raised from 29 to 64%.

## Supplementary Information

Below is the link to the electronic supplementary material.

Supplementary file1 (PDF 1157 kb)

## References

[1] Bronselaer B, Zanna L (2020). Heat and carbon coupling reveals ocean warming due to circulation changes. Nature.

[2] Schwartzman A, Keeling RF (2020). Achieving atmospheric verification of CO_2_ emissions. Nat. Clim. Change.

[3] Pan ZB, Han ES, Zheng JG, Lu J, Wang XL (2020). Highly efficient photoelectrocatalytic reduction of CO_2_ to methanol by a p-n heterojunction CeO_2_/CuO/Cu catalyst. Nano-Micro Lett..

[4] Jiao XC, Zheng K, Liang L, Li XD, Sun YF (2020). Fundamentals and challenges of ultrathin 2D photocatalysts in boosting CO_2_ photoreduction. Chem. Soc. Rev..

[5] Hao L, Huang HW, Zhang YH, Ma TY (2021). Oxygen vacant semiconductor photocatalysts. Adv. Funct. Mater..

[6] Chen F, Ma TY, Zhang TR, Zhang YH, Huang HW (2021). Atomic-level charge separation strategies in semiconductor-based photocatalysts. Adv. Mater..

[7] Yuan L, Geng ZY, Xu JK, Guo F, Han C (2021). Metal-semiconductor heterostructures for photoredox catalysis: where are we now and where do we go?. Adv. Funct. Mater..

[8] Zhang YZ, Xia BQ, Ran JR, Davey K, Qiao SZ (2020). Atomic-level reactive sites for semiconductor-based photocatalytic CO_2_ reduction. Adv. Energy Mater..

[9] Zhao XL, Chen S, Yin HJ, Jiang SY, Zhao K (2020). Perovskite microcrystals with intercalated monolayer MoS_2_ nanosheets as advanc-ed photocatalyst for solar-powered hydrogen generation. Matter.

[10] Kosco J, Bidwell M, Cha H, Martin T, Howells CT (2020). Enhanced photocatalytic hydrogen evolution from organic semiconductor heterojunction nanoparticles. Nat. Mater..

[11] Xia CK, Wang H, Kim JK, Wang JY (2021). Rational design of metal oxide-based heterostructure for efficient photocatalytic and photoelectrochemical systems. Adv. Funct. Mater..

[12] Zhao X, Luo WJ, Feng JY, Li MX, Li ZS (2014). Quan-titative analysis and visualized evidence for high charge separation efficiency in a solid-liquid bulk heterojunction. Adv. Energy Mater..

[13] Rettie AJE, Lee HC, Marshall LG, Lin JF, Capan C (2013). Combined charge carrier transport and photoelectrochemical characterization of BiVO_4_ single crystals: intrinsic behavior of a complex metal oxide. J. Am. Chem. Soc..

[14] Shi Q, Li Z, Chen L, Zhang X, Han W (2019). Synthesis of SPR Au/BiVO_4_ quantum dot/rutile-TiO_2_ nanorod array composites as efficient visible-light photocatalysts to convert CO_2_ and mechanism insight. Appl. Catal. B Environ..

[15] Hou Y, Chen X, Yang S, Li C, Zhao H (2017). A band-edge potential gradient heterostructure to enhance electron extraction efficiency of the electron transport layer in high-performance perovskite solar cells. Adv. Funct. Mater..

[16] Liu YP, Zhang SY, He J, Wang ZMM, Liu ZW (2019). Recent progress in the fabrication, properties, and devices of heterostructures based on 2D materials. Nano-Micro Lett..

[17] Jiang WS, Zong XP, An L, Hua SX, Miao X (2018). Consciously constructing heterojunction or direct Z-scheme photocatalysts by regulating electron flow direction. ACS Catal..

[18] Xu QL, Zhang LY, Cheng B, Fan JJ, Yu JG (2020). S-scheme heterojunction photocatalyst. Chem.

[19] Low JX, Yu JG, Jaroniec M, Wageh S, Al-Ghamdi AA (2017). Heterojunction photocatalysts. Adv. Mater..

[20] Starr MB, Shi J, Wang XD (2012). Piezopotential-driven redox reactions at the s-urface of piezoelectric materials. Angew. Chem. Int. Ed..

[21] Oishi M, Yamanaka K, Watanabe I, Shimoda K, Matsunaga T (2016). Direct observation of reversible oxygen anion redox reaction in Li-rich manganese oxide, Li_2_MnO_3_, studied by soft X-ray absorption spectroscopy. J. Mater. Chem. A.

[22] Zhan WW, Sun LM, Han XG (2019). Recent progress on engineering highly efficient porous semiconductor photocatalysts derived from metal-organic frameworks. Nano-Micro Lett..

[23] Low J, Jiang C, Cheng B, Wageh S, Al-Ghamdi AA (2017). A review of direct Z-scheme photocatalysts. Small Methods.

[24] Hu Y, Hao X, Cui Z, Zhou J, Chu S (2020). Enhanced photoca-rrier separation in conjugated polymer engineered CdS for direct Z-scheme phot-ocatalytic hydrogen evolution. Appl. Catal. B Environ..

[25] Chen X, Zhang Z, Chi L, Nair AK, Shangguan W (2016). Recent advances in visible-light-driven photoelectrochemical water splitting: catalyst nanostructures and reaction systems. Nano-Micro Lett..

[26] Xu Q, Zhang L, Yu J, Wageh S, Al-Ghamdi AA (2018). Direct Z-scheme photocatalysts: principles, synthesis, and applications. Mater. Today.

[27] Liu B, Wu CH, Miao JW, Yang PD (2014). All inorganic semiconductor nano-wire mesh for direct solar water splitting. ACS Nano.

[28] Jing LQ, Zhou W, Tian GH, Fu HG (2013). Surface tuning for oxide-based na-nomaterials as efficient photocatalysts. Chem. Soc. Rev..

[29] Mathew K, Sundararaman R, Weaver KL, Arias TA, Henn-ig RG (2014). Implicit solvation model for density-functional study of nanocrystal surfaces and reaction pathways. J. Chem. Phys..

[30] Mathew K, Kolluru VSC, Mula S, Steinmann SN, Hennig RG (2019). Implicit self-consistent electrolyte model in plane-wave density-functional theory. J. Chem. Phys..

[31] Perdew JP, Burke K, Ernzerhof M (1996). Generalized gradient approximation made simple. Phys. Rev. Lett..

[32] Wang L, Maxisch T, Ceder G (2006). Oxidation energies of transition metal oxides within the GGA+U framework. Phys. Rev. B.

[33] Grimme S, Antony J, Ehrlich S, Krieg H (2010). A consistent and accurate ab initio parametrization of density functional dispersion correction (DFT-D) for the 94 elements H-PU. J. Chem. Phys..

[34] Grimme S, Ehrlich S, Goerigk L (2011). Effect of the damping function in dispersion corrected density functional theory. J. Comput. Chem..

[35] Zheng M, Cao XH, Ding Y, Tian T, Lin JQ (2018). Boosting photocatalytic wa-ter oxidation achieved by BiVO_4_ coupled with iron-containing polyoxometalate: analysis the true catalyst. J. Catal..

[36] Yang JL, Sun N, Zhang ZQ, Bian J, Qu Y (2020). Ultrafine SnO_2_/010 facet-exposed BiVO_4_ nanocomposites as ef-ficient photoanodes for controllable conversion of 2,4-Dichlorophenol via a pref-erential dechlorination path. ACS Appl. Mater. Interfaces.

[37] Ma Y, Du YY, Chen Y, Gu CJ, Jiang T (2020). Intrinsic raman signal of polymer matrix induced quantitative multiphase sers analysis based on stretched PDMS film with anchored Ag nanoparticles/Au nanowires. Chem. Eng. J..

[38] Kang M, Kim JJ, Oh YJ, Park SG, Jeong KH (2014). A deformable nanopl-asmonic membrane reveals universal correlations between plasmon resonance andsurface enhanced raman scattering. Adv. Mater..

[39] Hu JQ, He HC, Zhou X, Li ZS, Shen Q (2019). BiVO4 tubular structures: oxygen defect-rich and largely exposed reactive 010 facets synergistically boost photocatalytic water oxidation and the selective N=N coupling reaction of 5-amino-1H-tetrazole. Chem. Commun..

[40] Dong CW, Lu SY, Yao SY, Ge R, Wang ZD (2018). Colloidal synthesis of ultrathin monoclinic BiVO_4_ nanosheets for Z-scheme overall water splitting under visible light. ACS Catal..

[41] Yao D, Dong CW, Bing QM, Liu Y, Qu FD (2019). Oxygen-defective ultrathin BiVO_4_ nanosheets for enhanced gas sensing. ACS Appl. Mater. Interfaces.

[42] Wang SC, He TW, Chen P, Du AJ, Ostrikov K (2020). In situ formation of oxygen vacancies achieving near-complete charge separ-ation in planar BiVO_4_ photoanodes. Adv. Mater..

[43] Jaihindh DP, Thirumalraj B, Chen SM, Balasubramanian P, Fu YP (2019). Facile synthesis of hierarchically nanostructured bismuth vanadate: an efficient photocatalyst for degradation and detection of hexavalent chromium. J. Hazard. Mater..

[44] Wu JM, Chen Y, Pan L, Wang PH, Cui Y (2018). Multi-layer monoclinic BiVO_4_ with oxygen vacancies and V^4+^ species for highly efficient visible-light photoelectrochemical applications. Appl. Catal. B Environ..

[45] Wang J, Osterloh FE (2014). Limiting factors for photochemical charge separation in BiVO_4_/Co_3_O_4_, a highly active photocatalyst for water oxidation in sunlight. J. Mater. Chem. A.

[46] Ghoneim MT, Zidan MA, Alnassar MY, Hanna AN (2015). Thin PZT-based ferroelectric capacitors on flexible sili-con for nonvolatile memory applications. Adv. Electron. Mater..

[47] Xie MZ, Fu XD, Jing LQ, Luan P, Feng YJ (2014). Long-lived, visible-light-excited charge carriers of TiO_2_/BiVO_4_ nanocomposites and their une-xpected photoactivity for water splitting. Adv. Energy Mater..

[48] Wang SC, Chen P, Yun JH, Hu YX, Wang LZ (2017). An electrochemically treated BiVO_4_ photoanode for efficient photoelectrochemical water splitting. Angew. Chem. Int. Ed..

[49] Shi QJ, Zhang M, Zhang ZM, Li YX, Qu Y (2020). Energy and separation optimization of photogenerated charge in BiVO_4_ quantum dots by piezo-potential for efficient gaseous pollutant degradation. Nano Energy.

[50] Antony RP, Bassi PS, Abdi FF, Chiam SY, Ren Y (2016). Electrospun Mo-BiVO_4_ for efficient photoelectrochemical water oxidation: Direct evidence of improved hole diffusion length and charge separation. Electrochim. Acta.

[51] Yang XL, Wang SY, Yang N, Zhou W, Wang P (2019). Oxygen vacancies induced special CO_2_ adsorption modes on Bi_2_MoO_6_ for highly selective conversion to CH_4_. Appl. Catal. B Environ..

[52] Xing MY, Zhou Y, Dong CY, Cai LJ, Zeng LX (2018). Modulation of the reduction potential of TiO_2_-x by fluorination for efficient and selective CH_4_ generation from CO_2_ photoreduction. Nano Lett..

