# Supplementary material for: Electrostatic Field Enhanced Photocatalytic CO2 Conversion on BiVO4 Nanowires
Source: Nanomicro Lett. 2021 Dec 6;14:15. doi: 10.1007/s40820-021-00749-6 (PMC8649055; doi:10.1007/s40820-021-00749-6)
Supplement: Supplementary file 1 — Supplementary file1 (PDF 1157 kb) [file 40820_2021_749_MOESM1_ESM.pdf]

Supporting Information for

Electrostatic Field Enhanced Photocatalytic CO_2 Conversion on $BiVO_4$ Nanowires

Shuai Yue[1,#], Lu Chen[1,#], Manke Zhang[1], Zhe Liu[1], Tao Chen[2], Mingzheng Xie[1,*], Zhen Cao[2,*], Weihua Han[2,*]

[1]Key Laboratory for Environmental Pollution Prediction and Control of Gansu Province, College of Earth and Environmental Sciences, Lanzhou University, Lanzhou 730000, P. R. China

[2]School of Physical Science and Technology, Lanzhou University, Lanzhou 730000, P. R. China

[#]Shuai Yue and Lu Chen contributed equally to this work

*Corresponding authors. E-mail: xiemzh@lzu.edu.cn (Mingzheng Xie); caozhen@lzu.edu.cn (Zhen Cao); hanwh@lzu.edu.cn (Weihua Han)

S1 Experimental Calculations

S1.1 Calculation of the Incident Photon-to-Current Efficiency (IPCE)

Incident photon-to-current efficiency (IPCE) utilized light from a 300 W Xe lamp passed through a monochromator, and recorded LSV using electrochemical workstation. The incident light interval was 10 nm from 400 nm to 600 nm, and it was calibrated by a standard Newport Si PV module [S1]. The equation used to obtain the IPCE values was:

$$\text{IPCE} = \frac{\text{electrons}}{\text{photons}} = \frac{J_{ph} \times 1239.8}{P_{mono} \times \lambda}$$

Among them, J_{ph} was the photocurrent density minus the dark current density, and P_{mono} was determined by calibrated Newport Si PV module. λ was the Incident light wavelength [S2]. For example, the IPCE at 400 nm under 0 V could be calculated as follows:

$$\text{IPCE} = \frac{J_{ph} \times 1239.8}{P_{mono} \times \lambda} = \frac{(0.0190334981) \times 1239.8}{\frac{0.0124}{0.227 \times 0.25} \times 400} \times 100\% = 26.99\%$$

S1.2 Calculation of the Apparent Quantum Yields (AQE)

The apparent quantum yields (AQE) were calculated from the incident light intensity and generation rate of CH_4 and CO according the equation: AQE= (8×number of evolved CH_4 molecules+2×number of evolved CO molecules) /number of incident photons= $(8\times R_1+2\times R_2) N_A hc/AP\lambda$ [S3], in which R_1 and R_2 were the generation rate of CH_4 and CO; N_A was Avogadro constant; h and c were the Planck constant and light speed; P and λ were the intensity and the wavelength of incident light, respectively. The used light source was a 500 W Xenon lamp equipped with a bandpass filter, and BVO-NWs/PDMS/PZT was used as photocatalyst. The area of incident light (A) was 9π cm^2, which was determined by the size of filter. The light

intensity was determined by means of standard silicon solar cell. The detailed information about intensity of incident light and corresponding generation amount of CH_4 and CO were shown in Table S1. Hence, its AQE value at 400 nm could be calculated as follows:

$$AQE_{(400nm)} = \frac{(8\times R_1+2\times R_2)\times N_A\times h\times c}{AP\lambda}$$

$$AQE_{(400nm)}=\frac{[(8\times 15.94+2\times 9.02)\times 10^{-6}/(8\times 3600)]\times 6.02\times 6.626\times 3\times 10^{-3}}{9\pi\times 10^{-4}\times(9.02\times 10^{-3}/10^{-4})\times 400\times 10^{-9}}\times 100\%=0.59\%$$

S1.3 Calculation of the Charge Carrier Diffusion Lengths (L)

The charge carrier diffusion lengths (L) of BVO-NWs/PDMS/PZT were estimated with Gartner model [S4] and the equations were as follows:

$$\frac{1}{C^2} = \frac{2}{\varepsilon\varepsilon_0 A^2 e N_d}(V-V_{fb}-\frac{k_B T}{e})\cdots\cdots\cdots(S1)$$

$$W = \sqrt{\frac{2\varepsilon_o\varepsilon(V-V_f)}{qN_d}}\cdots\cdots\cdots\cdots\cdots\cdots\cdots\cdots(S2)$$

Where C was the space charge layer capacitance, ε was the relative permittivity of $BiVO_4$ [S5], ε_0 was the vacuum permittivity, A was the electrode exposed area, e was electronic charge, N_d was the charge carrier density, V was the applied potential, V_{fb} was the flat band potential, k_B was the Boltzmann constant, T was the temperature. According to the Mott-Schottky plots (Fig. 5b) and Eq. (S1), the charge carrier density can be calculated. W was the depletion width and it can be calculated by Eq. (S2).

$$L = \frac{\frac{e^{-\alpha W}}{1-\eta_{sep}}-1}{\alpha}\cdots\cdots\cdots\cdots\cdots\cdots\cdots\cdots\cdots\cdots(S3)$$

Where L was the charge carrier diffusion lengths, η_{sep} was the quantum efficiency which was determined by APCE in 0.5 M Na_2SO_3. α was the absorption coefficient at 400 nm. The calculation results were shown in **Tab. S4**.

S2 Supplementary Figures and Tables

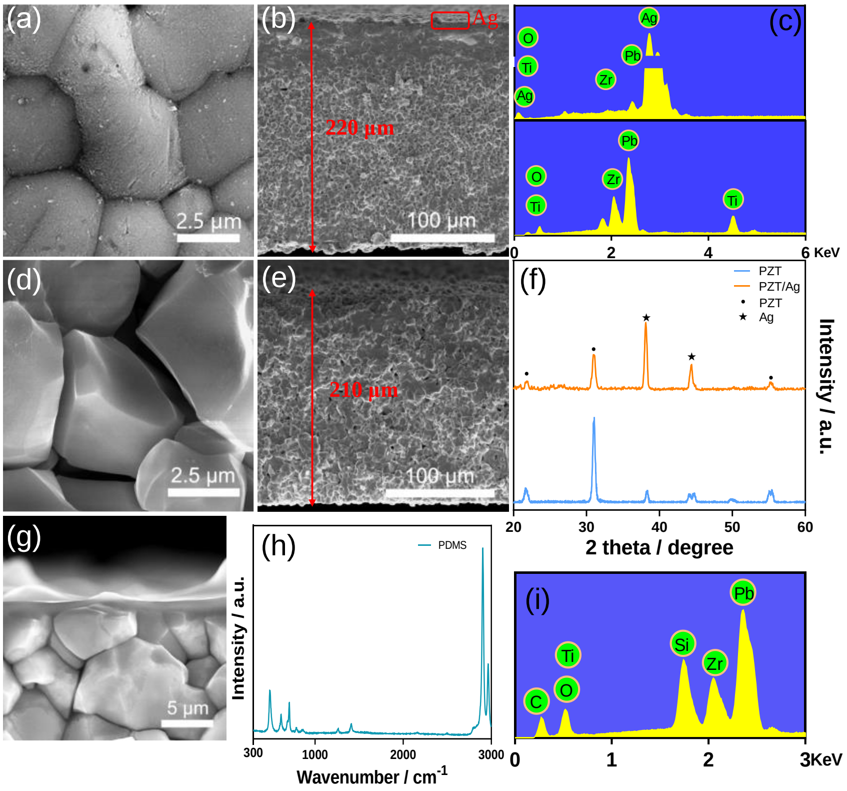

Fig. S1 SEM images of PZT with silver coating on the surface (**a, b**) and silver coating removed (**d, e**). The corresponding EDX spectra (**c**) and XRD pattern (**f**). SEM image (**g**), Raman spectra (**h**) and EDX spectra (**i**) of PDMS layer

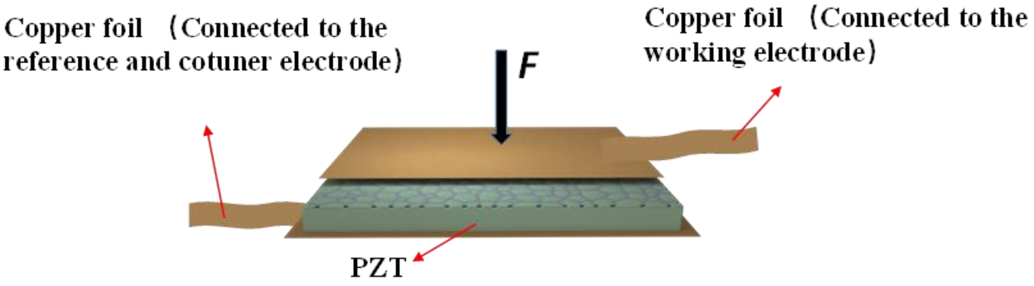

Fig. S2 Two-electrode structure for piezoelectric measurement

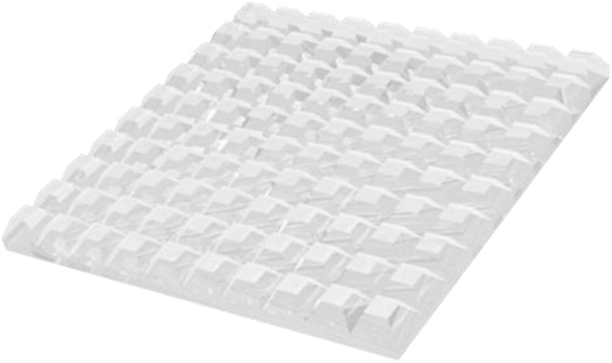

Fig. S3 The diagram of covered quartz glass

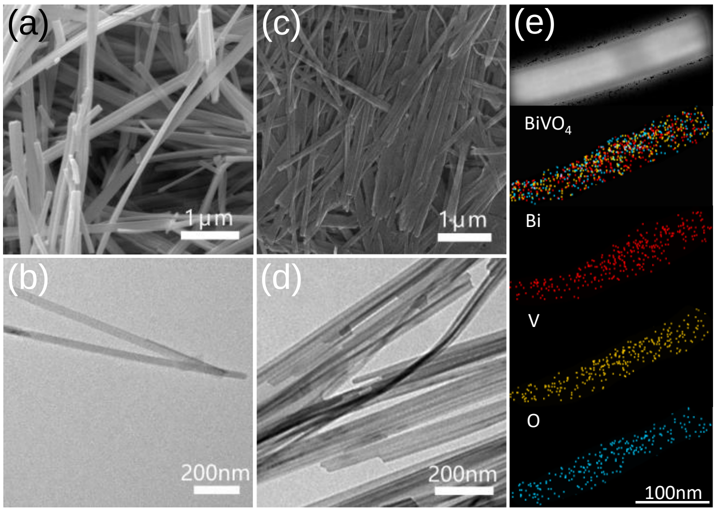

Fig. S4 SEM and TEM images of $BiVO_4$ nanowires (**a, b**) and $Na_2V_6O_{16}\cdot 3H_2O$ nanowires (**c, d**). The elemental maps of $BiVO_4$ nanowires (**e**)

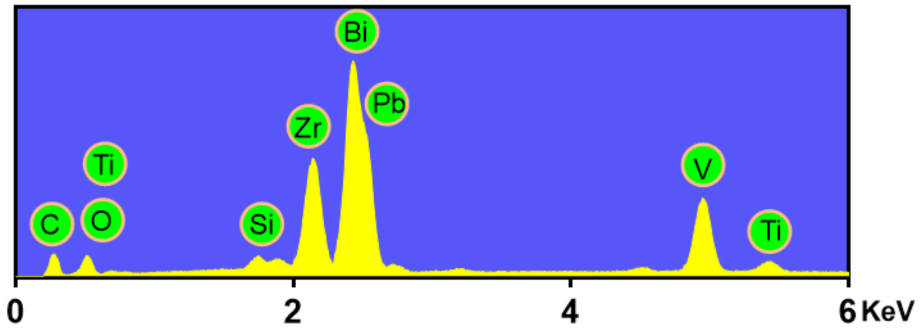

Fig. S5 EDX spectra of BVO-NWs/PDMS/PZT

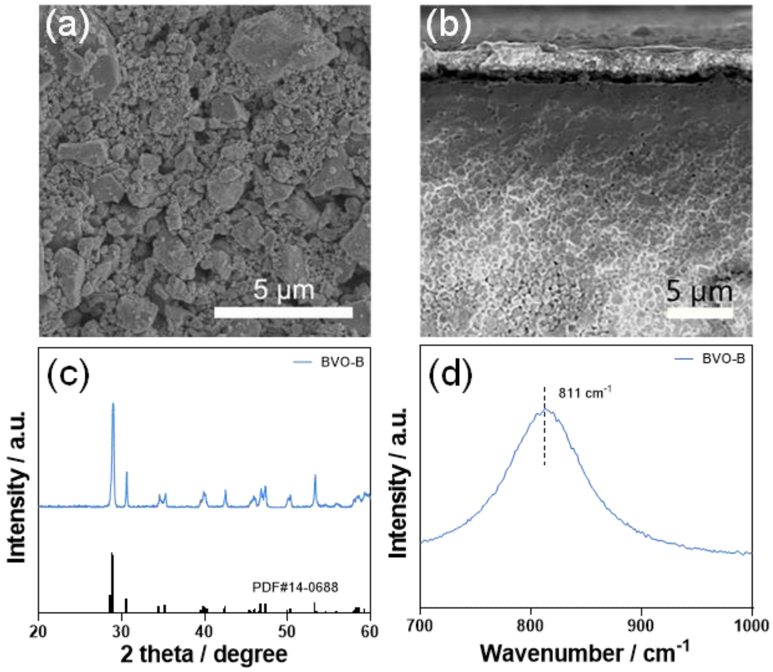

Fig. S6 SEM images (**a-b**), XRD pattern (**c**) and Raman spectrum (**d**) of the bulk $BiVO_4$. The bulk $BiVO_4$ particles were prepared by a hydrothermal method and were denoted as BVO-B [S6].

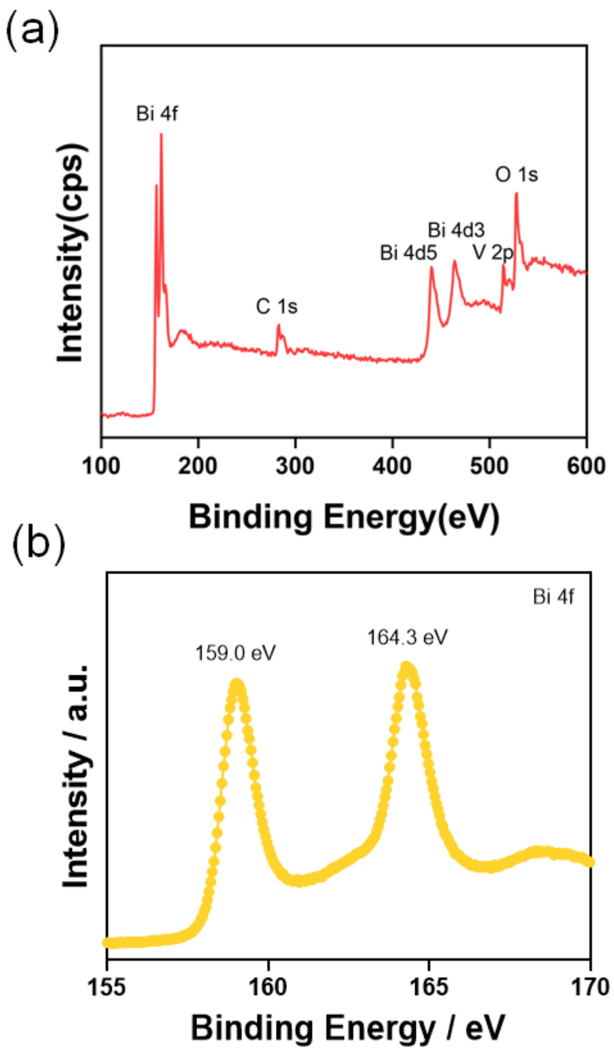

Fig. S7 XPS survey spectrum (**a**) and high resolution XPS spectra (**b**) of $BiVO_4$ nanowires

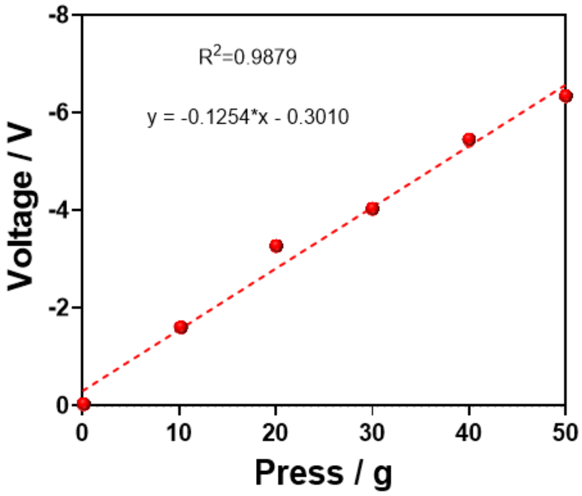

Fig. S8 The relationship between stress and piezoelectric potential

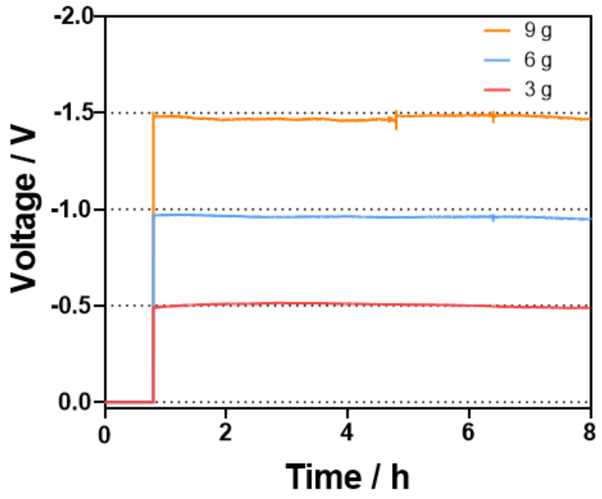

Fig. S9 Long time piezoelectric performances of PZT substrate under different stresses

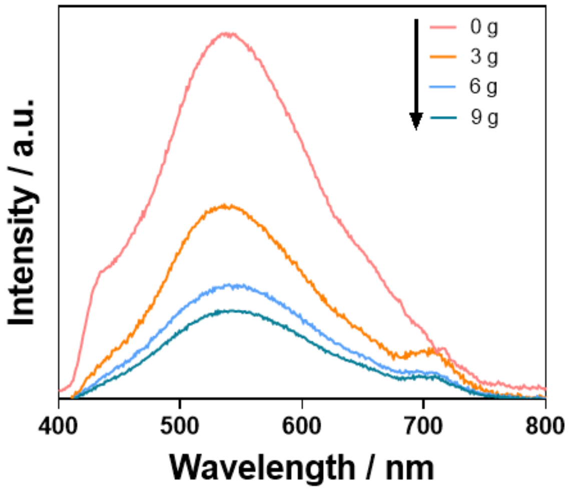

Fig. S10 The photoluminescence spectra of BVO-NWs/PDMS/PZT under different stresses

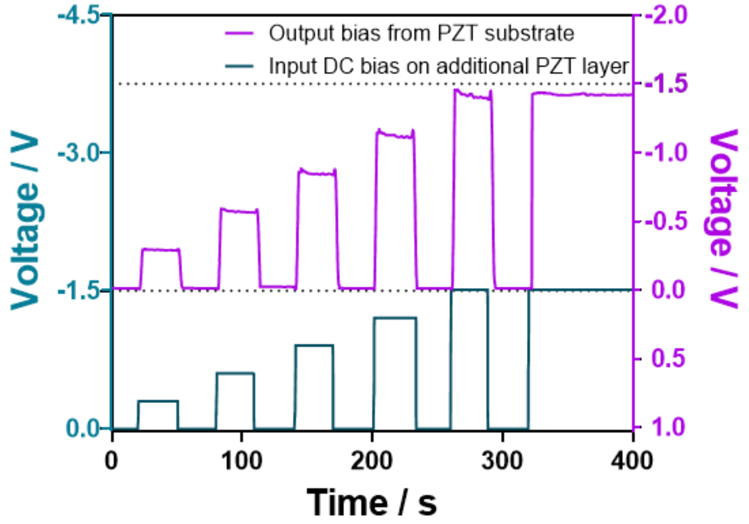

Fig. S11 The dependence of output bias from PZT substrate on the applied DC bias on additional PZT layer

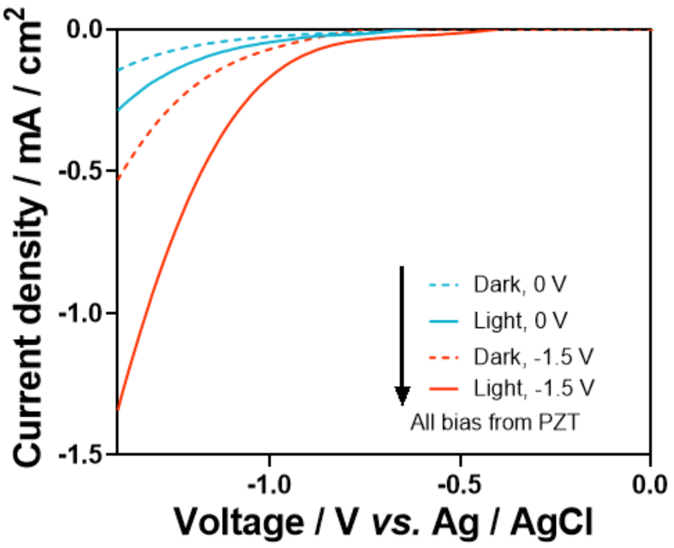

Fig. S12 *I-V* curves of BVO-NWs in CO_2-bubbled system

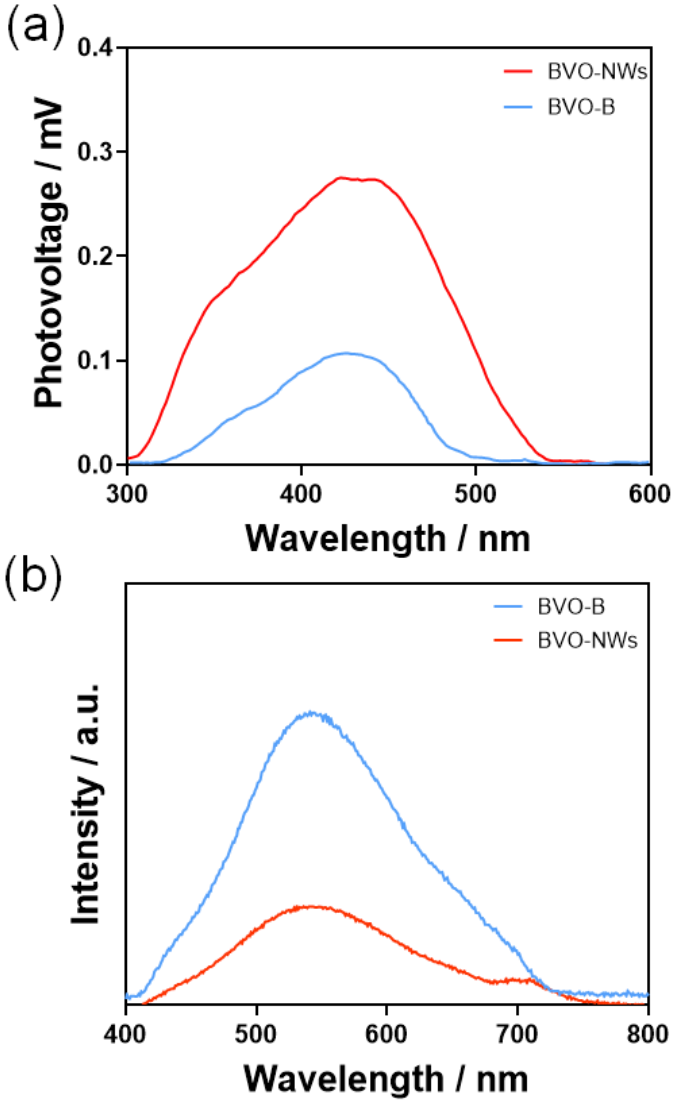

Fig. S13 The surface photovoltage spectra (**a**) and photoluminescence spectra (**b**) of samples

Table S1 The incident light intensity and amount of generated CH_4 and CO in 8 h of BVO-NWs/PDMS/PZT with different wavelengths

	$\lambda_{\pm 10}$ (nm)					
	400	440	480	520	560	600
Intensity of incident light (mW/cm^2)	9.02	9.02	11.75	11.75	11.54	10.28
Amount of generated CH_4(μmol)	15.94	15.73	13.00	10.91	3.15	1.89
Amount of generated CO (μmol)	9.02	9.02	7.55	6.08	1.68	1.05

Table S2 The comparative study of $BiVO_4$ NWs sample on photocatalytic CO_2 reduction with other related samples reported

Photocatalyst	Light Source Used	Activity	Sample Quality	Refs.
$BiVO_4$ microplates	300 W Xe lamp	CO: 0.58 μmol/g/h<br>CH_4: -	10 mg	[S7]
$BiVO_4$ nanosheets	300 W Xe lamp (λ>420 nm)	CO: 0.29 μmol/g/h<br>CH_4: 0.30 μmol/g/h	50 mg	[S8]
$BiVO_4$ octahedron	300 W Xe lamp (λ>420 nm)	CO: 0.45 μmol/g/h<br>CH_4: 0.59 μmol/g/h	100 mg	[S9]
$BiVO_4$ microspheres	300 W Xe lamp	CO: 1.3 μmol/g/h<br>CH_4: -	10 mg	[S10]
$BiVO_4$ nanoparticles	300 W Xe lamp (λ>420 nm)	CO: 0.30 μmol/g/h<br>CH_4: -	50 mg	[S11]
**$BiVO_4$ nanowires**	**300 W Xe lamp**	**CO: 0.76 μmol/cm²/h**<br>**CH_4: 0.32 μmol/cm²/h**	**3.15 cm² (~2 mg)**	**This work**

Table S3 The charge carriers lifetimes of sample under different piezo bias

Bias from PZT	f_{max} (Hz)	lifetime of charge carrier (μs)
0.0 V	33.11	4.8
-0.5 V	14.13	11.3
-1.0 V	8.32	19.1
-1.5 V	2.51	63.4

Table S4 The carrier concentrations and hole diffusion lengths of sample under different piezo bias

Bias from PZT	V_f (V *vs.* SHE)	N_d (cm^{-3})	W (nm)	η_{sep}	L_D (nm)
0.0 V	-0.61	5.13×10^{19}	15.37	0.2080	39.26
-0.5 V	-0.67	7.97×10^{19}	12.56	0.3472	79.51
-1.0 V	-0.75	1.18×10^{20}	10.58	0.4541	124.37
-1.5 V	-0.81	1.52×10^{20}	9.47	0.5070	153.72

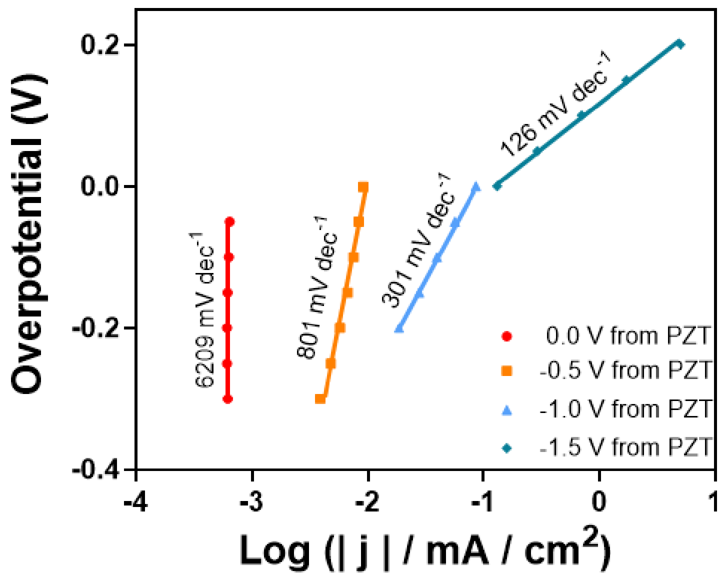

Fig. S14 Tafel slopes of sample under different bias

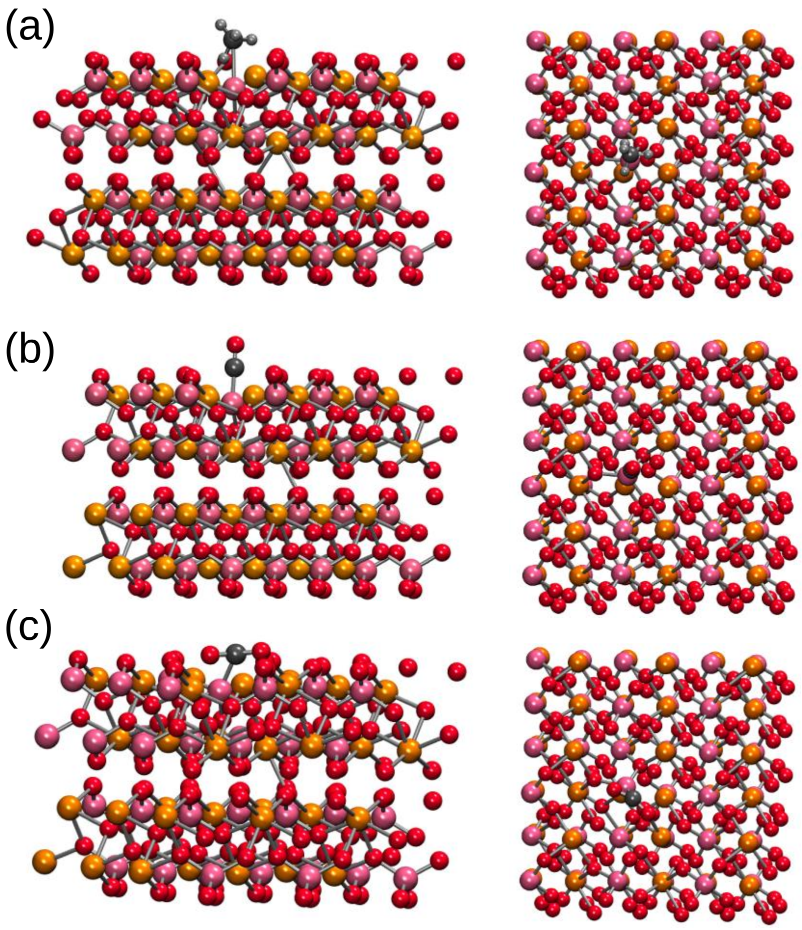

Fig. S15 CH_4 (**a**), CO (**b**) and CO_2 (**c**) gas molecules adsorbed on $BiVO_4$ layer

Supplementary References

[S1] J. A. Seabold, K. Zhu, N. R. Neale, Efficient solar photoelectrolysis by nanoporous Mo: $BiVO_4$ through controlled electron transport. Phys. Chem. Chem. Phys. **16**(3), 1121-1131 (2014). https://doi.org/10.1039/c3cp54356k

[S2] P. R. F. Barnes, B. C. O'Regan, Electron recombination kinetics and the analysis of collection efficiency and diffusion length measurements in dye sensitized solar cells. J. Phys. Chem. C **114**(44), 19134-19140 (2010). https://doi.org/10.1021/jp106329a

[S3] L. Chen, C. Hou, Z. Q. Liu, Y. Qu, M. Z. Xie et al., Inhibition of Sn(ii) oxidation in Z-scheme $BiVO_4$-QD@Sn_3O_4 for overall water splitting. Chem. Commun. **56**(89), 13884-13887 (2020). https://doi.org/10.1039/d0cc05566b

[S4] R. P. Antony, P. S. Bassi, F. F. Abdi, S. Y. Chiam, Y. Ren et al., Electrospun Mo-$BiVO_4$ for efficient photoelectrochemical water oxidation: Direct evidence of improved hole diffusion length and charge separation. Electrochim. Acta **211**, 173-182 (2016). https://doi.org/10.1016/j.electacta.2016.06.008

[S5] S. C. Wang, P. Chen, J. H. Yun, Y. X. Hu, L. Z. Wang, An electrochemically treated $BiVO_4$ photoanode for efficient photoelectrochemical water splitting. Angew. Chem. Int. Ed. **56**(29), 8500-8504 (2017). https://doi.org/10.1002/anie.201703491

[S6] M. Z. Xie, Z. M. Zhang, W. H. Han, X. W. Cheng, X. L. Li et al., Effi-cient hydrogen evolution under visible light irradiation over $BiVO_4$ quantum dot decorated screw-like SnO_2 nanostructures. J. Mater. Chem. A **5**(21), 10338-10346 (2017). https://doi.org/10.1039/c7ta01415e

[S7] L. Huang, Z. Y. Duan, Y. Y. Song, Q. S. Li, L. M. Chen, $BiVO_4$ microplates with oxygen vacancies decorated with metallic Cu and Bi nanoparticles for CO_2 photoreduction. ACS Appl. Nano Mater. **4**(4), 3576-3585 (2021). https://doi.org/10.1021/acsanm.1c00115

[S8] J. Bian, J. Feng, Z. Zhang, Z. Li, Y. Zhang et al., Dimension-matched zinc phthalocyanine/$BiVO_4$ ultrathin nanocomposites for CO_2 reduction as efficient wide-visible-light-driven photocatalysts via a cascade charge transfer. Angew. Chem. Int. Ed. **58**(32), 10873-10878 (2019). https://doi.org/10.1002/anie.201905274

[S9] C. G. Zhou, S. M. Wang, Z. Y. Zhao, Z. Shi, S. C. Yan et al., A facet-dependent Schottky-junction electron shuttle in a $BiVO_4$\{010\}-Au-Cu_2O Z-scheme photocatalyst for efficient charge separation. Adv. Funct. Mater. **28**(31), 1801214 (2018). https://doi.org/10.1002/adfm.201801214

[S10] Z. Duan, X. Zhao, C. Wei, L. Chen, Ag-Bi/$BiVO_4$ chain-like hollow microstructures with enhanced photocatalytic activity for CO_2 conversion. Appl. Catal. A: Gen. **594**, 117459 (2020). https://doi.org/10.1016/j.apcata.2020.117459

[S11] C. Kim, K. M. Cho, A. Al-Saggaf, I. Gereige, H. T. Jung, Z-scheme photocatal-ytic CO_2 conversion on three-dimensional $BiVO_4$/carbon-coated Cu_2O nanowire a-rrays under visible light. ACS Catal. **8**(5), 4170-4177 (2018). https://doi.org/10.1021/acscatal.8b00003
